# The development and validation of an emotional vulnerability scale for university students

**DOI:** 10.3389/fpsyg.2022.941250

**Published:** 2022-09-15

**Authors:** Shinji Yamaguchi, Yujiro Kawata, Yuka Murofushi, Tsuneyoshi Ota

**Affiliations:** ^1^Faculty of Health and Sports Science, Juntendo University, Chiba, Japan; ^2^Graduate School of Health and Sports Science, Juntendo University, Chiba, Japan; ^3^Institute of Health and Sports Science and Medicine, Juntendo University, Chiba, Japan

**Keywords:** vulnerability, mental health, university students, scale development, depression

## Abstract

This study developed an emotional vulnerability scale and examined its reliability and validity with a sample of university students. In health psychology, a measurement of emotional pain (“hurt feelings”) can contribute to the prevention and improvement of physical and mental health problems in daily life. We collected data from 361 Japanese university students (186 men and 175 women; mean age = 19.6 ± 0.98 years). From preliminary interviews with 20 participants, 42 semantic units were extracted. For scale development, a questionnaire survey was conducted using the 42 extracted categories, and exploratory and confirmatory factor analyses were performed. Four factors (16 items) emerged, which were both reliable and valid: (1) “vulnerability toward criticism or denial,” (2) “vulnerability toward worsening relationships,” (3) “vulnerability toward interpersonal discord,” and (4) “vulnerability toward procrastination and emotional avoidance.” This scale can be useful to understand vulnerability in everyday situations and grasp the vulnerable conditions experienced by individuals. This can help prevent stress responses (such as depression and sadness) and mental health problems, which are valuable contributions to health psychology.

## Introduction

Emotional pain is part of the human experience, although its causes may vary. Characterized by Leary et al. (1998) as “hurt feelings,” emotional pain is likely to be perceived as physical pain (Chen et al., 2008). People at a high risk of emotional pain are described as psychologically vulnerable (Jose, 2005). It is possible for some people to be more susceptible to emotional pain than others. The phenomenon of being at risk for psychological hurt is referred to as “vulnerability,” which was proposed by Sinclair and Wallston (1999) as a psychological construct. They defined it as a “pattern of cognitive beliefs reflecting dependence on achievement or external sources of affirmation for one’s sense of self-worth” (Sinclair and Wallston, 1999, p. 120). From their definition, external sources can indicate events in daily life and interpersonal relationships that may elicit varying degrees of emotional pain, mediated by negative cognitive styles. How one responds to external sources of information depends on how one perceives events that occur in daily life and interpersonal relationships. Therefore, the more vulnerable people are, the more negative their perception of external sources, thus indicating how emotional pain is mediated by negative cognitive styles.

University students, who are the focus of this study, face many stressful life events, such as changes in their lifestyle, community, and relationships (Steptoe et al., 2007; Bayram and Bilgel, 2008; Ibrahim et al., 2013), thereby increasing their psychological vulnerability. Furthermore, maladaptive cognitive reactions to interpersonal events can affect coping behaviors, interpersonal relationships, and psychological and physical well-being (Nogueira et al., 2017). Thus, we developed an emotional vulnerability scale and examined its reliability and validity with a sample of university students.

## Vulnerability

Hayashi (2002) defined vulnerability in the Japanese context as a susceptibility to psychological harm and a possible state of fragility or emotional hurt. It represents a cognitive belief or experience that renders a person susceptible to hurt feelings in response to everyday life events. Additionally, vulnerability has been defined as a “cognitive structure that makes individuals more fragile under stressful environments, assuming that some people are more affected by stressful events than others” (Sinclair and Wallston, 1999, 2010). These definitions indicate that vulnerability is a “cognitive belief” (Çutuk and Aydoğan, 2019) about oneself being easily hurt and is distinct from personality traits or states. Thus, some people may be vulnerable if they have strong cognitive beliefs about themselves as being weak or fragile. Vulnerability is negatively associated with positive emotions, life satisfaction, and optimism (Sinclair and Wallston, 1999). Furthermore, vulnerable individuals display a negative interpretation of life events only when they are confronted with certain stressors, which places them at high risk for depression and other diverse negative outcomes (Ingram and Luxton, 2005). Increased vulnerabilities also lead to poor mental health and lack of social support (Akın and Eker, 2011). The negative correlation between vulnerability and self-efficacy has an adverse effect on behavioral motivation (Kiamarsi and Abolghasemi, 2014), and higher levels of vulnerability are associated with higher levels of depressive symptoms (Yamaguchi et al., 2018, 2019). Notably, women are more vulnerable than men (Yamaguchi et al., 2019). Furthermore, studies of athletes report that when vulnerable athletes engage in stress coping, they may focus on resolving emotional hurt and negative feelings or seek help from a reliable person (Yamaguchi et al., 2022). From a psychopathological perspective, vulnerability is associated with depression and anxiety disorders. Several cognitive vulnerability–stress models propose that one’s characteristic way of attending to, interpreting, and remembering negative events affects one’s psychopathological vulnerability (Reardon and Williams, 2007). Therefore, severe vulnerability may lead to mental illness. These findings show that vulnerability is significantly associated with negative attitudes that impede mental recovery and that vulnerability affects not only human mental health, but also interpersonal relationships and behavior.

### Measures of vulnerability

Several measures have been developed to assess vulnerability, some of which have been used by the studies previously mentioned. The psychological vulnerability scale (PVS) was developed by Sinclair and Wallston (1999) based on the emotional difficulties experienced by rheumatoid arthritis patients. According to Sinclair and Wallston (1999, 2), the PVS was designed to identify individuals with cognitive patterns that make them more susceptible to stress. Vulnerability in this study was defined according to the notion of “cognitive belief” mentioned earlier. Specific items include: “If I do not achieve my goals, I feel like a failure as a person,” “I am frequently aware of feeling inferior to other people,” and “I need approval from others to feel good about myself.” From such items, it is possible to understand how effectively patients with rheumatoid arthritis have adapted to the pain and dysfunction associated with their condition. Using this scale, screening can be performed for cognitive vulnerability related to perceptions of dependency, perfectionism, negative attributions, and the need for external sources of approval. The athletic vulnerability scale (AVS) was developed by Yamaguchi et al. (2019) to determine athletes’ susceptibility to emotional hurt in sports settings. This scale, which was developed in Japan, was based on the definition of Hayashi (2002), with reference to Sinclair and Wallston’s PVS and related research. Specific items include: “I feel emotionally hurt when peers talk about me behind my back;” “During games, I feel depressed if I do not produce my usual performance;” and “I lose confidence if athletes of a lower level than me receive good evaluations.” As these items show, terms specific to the target population are used, such as “match,” “competition,” and “athlete.”

In addition, one factor of a 134-item self-descriptive inventory developed by Altman and Wittenborn (1980) listed items related to narcissistic vulnerability, such as “I cry immediately” and “I am very sensitive to being criticized.” Although it is possible to assess the vulnerability of an individual as a personality trait, these items do not constitute scales, but represent a single factor assessed by the scale. Furthermore, it is possible that vulnerability connected with self-described dysphoria is different from the original state of vulnerability. In connection with the above Crowe et al. (2018), developed the Narcissistic Vulnerability Scale, a questionnaire that rates adjectives such as “Ashamed,” “Ignored,” “Underappreciated,” and “Vengeful.” We posit that these items are not related to vulnerability but are measures of psychiatric narcissism; therefore, it is difficult to determine whether they truly measure an individual’s vulnerability. Thus, vulnerability measures that have been developed to date are limited to specific groups of people or individual personality traits.

Schaufeli et al. (2002) pointed out the need to create measurement indicators that are tailored to the attributes and characteristics of the participants and to perform evidence-based analysis based on accurate psychological assessment. Therefore, Sinclair and Wallston (1999) and Yamaguchi et al. (2019) created measures of vulnerability specifically for patients with rheumatoid arthritis and university athletes, respectively, thereby addressing Schaufeli et al. (2002) point. However, using the above scales, it may be difficult to measure vulnerable conditions and emotional pain in interpersonal relationships, such as those experienced in daily life. For example, the AVS (Yamaguchi et al., 2019) cannot be used to measure the vulnerability of university students in daily life. In addition, a scale measuring the vulnerable conditions specifically experienced by patients with rheumatoid arthritis may not be suitable for measuring the experience of vulnerability in healthy participants who are responding to everyday difficulties in life and interpersonal relationships. To date, no indicators have been developed to measure the events and conditions related to vulnerability that people in general commonly experience in daily life. An emotional vulnerability scale that can be universally applied without limiting its scope to a target audience or specific situation is yet to be developed. In addition, it is unclear whether measuring vulnerability in relation to self-described dysphoria truly captures the concept of vulnerability. Therefore, it is difficult to measure an individual’s vulnerability in everyday situations, which seems to be an issue common to all the scales mentioned above.

In sum, existing measures of vulnerability tend to focus on specific populations, such as athletes or people with rheumatoid arthritis. Thus, we developed an emotional vulnerability scale to establish the degree of vulnerability experienced by university students concerning everyday situations and events that may be applicable to even non-athletic students and healthy individuals. According to Leary et al. (1998) and Feeney (2005), “hurtful experiences” readily occur for many people on a daily basis, are memorable for a long period, and can have a significant impact on human cognition and behavior. In that context, university students interact and form close relationships with more people than junior high school and high school students and experience several life events related to their future prospects. Consequently, highly vulnerable people may respond with intense hurt feelings to specific events, which may impair their mental health. The specific conditions vary across individuals. Being able to measure and understand the vulnerability experiences and conditions of university students may elucidate their mental health.

## Preliminary investigation

The purpose of the preliminary investigation was to conduct a survey using semi-structured interviews among university students, gather data regarding factors that characterize vulnerability in daily life, and create a draft scale based on the collected data.

### Method of preliminary investigation

#### Participants

The participants for the preliminary study were 20 Japanese university students (10 men and 10 women; mean age = 21.1 ± 0.83 years). Included participants were active university students who had not taken a leave of absence owing to health-related, academic, or financial reasons in the past year. Students who had been medically diagnosed with mental disorders were excluded. Based on these inclusion and exclusion criteria, we used random sampling to recruit interview participants. To collect data on a wide range of vulnerability-inducing events that can be experienced in everyday situations, we assumed a required sample size of about 10 people. Twenty students volunteered to participate, none of whom were excluded.

#### Procedure

The survey was conducted from December 2020 to January 2021 using an online conference system. The interview consisted of three predetermined questions. Since physical pain was not the subject of this study, the questions were focused on experiences of emotional hurt. Shinmura (2018) states that vulnerability means feeling “fragile and weak.” When conducting an interview survey, we observed some difficulty among the participants in answering the question “Have you ever felt vulnerable?” Therefore, the question was split into “Have you ever felt fragile” and “Have you ever felt weak?” and participants’ responses were collected. The time required to complete each interview was approximately 30 min. The content of the interviews was recorded by an online conference system with the consent of the participants. As the survey was conducted online, we paid close attention to ensuring privacy; to do so, the first author and researchers used the conference room in the university, and the participants responded to the interview in a place that ensured privacy (e.g., home, alone in a room). Before interview commencement, we confirmed that the participants were alone.

We created the following questions for the semi-structured interview.

1. Have you ever experienced emotional hurt in your daily life? Can you explain more about it?
2. Have you ever felt “fragile” in your daily life? Specifically, what kind of event caused this feeling?
3. Have you ever felt “weak” in your daily life? Specifically, what kind of event caused this feeling?

#### Ethical considerations

Before the interview survey, participants were informed, in writing and verbally, about the purpose of the survey, that participation was voluntary, and that they would not be disadvantaged if they did not participate in the survey. In addition, they were informed that the recordings of the interviews would not be used for purposes other than that of this study. Participation in the study was considered as consent. This study was conducted with the approval of the institutional review board of the institution to which the principal author is affiliated. The specific approval number was “2020–15.”

#### Data analysis

The recorded interview data provided by the participants were transcribed. The data were sorted according to the Kawakita Jiro method (Kawakita, 1967). The vocabulary items were aggregated from similar expressions and categorized with labels indicating vulnerability in everyday situations, in accordance with Hayashi (2002, p. 1) definition of vulnerability as “a susceptibility to psychological harm, a possible state of brittle or emotional hurt.” Based on these items, the appropriate question items were created in Japanese. Regarding the aggregated vocabulary, items having ambiguous content or unclear meanings, which significantly differed from the definition of vulnerability, were excluded from the analysis. A university faculty member specializing in health psychology, another member specializing in sports psychology, a graduate student specializing in sports psychology, and another specializing in mental health science participated in a discussion on item selection. The four experts agreed verbally after examining the printed data from the interviews, and any disagreements were resolved through appropriate discussion. This corresponds to the concept of “Showing Face Validity” mentioned by Wood and Boyce (2017), affirming people’s views that the items are logical and relevant.

### Preliminary survey results and discussion

From the analysis performed on the preliminary data, we identified seven major categories: remorse, diluted relationship, pressure, difficulty to refuse, procrastination, avoidance/escape, and susceptibility to critique. In addition, 42 subcategories were identified based on an evaluation of the aggregated vocabulary and Hayashi’s definition of vulnerability (details of the categories and subcategories are presented in Supplementary Table 1).

#### Remorse

“Remorse” refers to content that expresses discouragement based on personal incompetence and the poor execution of an event. Answers regarding emotionally hurtful events that expressed remorse included “I feel inferior compared to others,” “It will hurt if you show your feelings on your face,” and “I will carry my mistakes around forever.” Remorse was expressed when participants had experienced emotionally hurtful events that they blamed themselves for.

#### Diluted relationship

“Diluted relationship” refers to instances where participants expressed being emotionally hurt by interpersonal relationships. Answers included “I do not get on well with friends,” “I feel like I am out of place,” and “The other party’s reply is slow.” The obtained content clearly showed participants’ vulnerability to psychological damage.

#### Pressure

“Pressure” refers to being emotionally hurt by tension and excessive anxiety. Responses such as, “I feel sick when I am responsible for something,” “Speaking in front of many people makes me nervous,” and “I am scared to fail,” among others, showed that participants experienced emotional pain in tense situations.

#### Difficulty to refuse

“Difficulty to refuse” refers to having trouble in actively declining a request or invitation from others; it describes the emotional pain the participant experiences when refusing a request. The inability to explicitly refuse a request or invitation indicates weakness or fragility. Responses such as “I cannot argue/I cannot oppose,” “I cannot refuse/I am pitiful if I cannot refuse,” and “I do not want to be disliked/I cannot decline an invitation” clearly showed participants’ susceptibility to interpersonal damage.

#### Regret over procrastination

“Regret over procrastination” refers to feelings of regret or remorse caused by the participant’s negligence. Answers included “I do nothing when I am alone,” “I am tired and cannot do that/I do not want to do it/I have to do it, but I do not,” and “I do not want to do anything.” The content expressed participants’ feelings of disgust toward themselves, conveying a sense of hurt.

#### Avoidance/escape

“Avoidance/escape” refers to avoidance of emotional hurt by escaping an event. Responses such as “I want to run away/It is not convenient for me,” “I will procrastinate/I give up,” and “I cannot keep it going” showed that, rather than taking action and experiencing pain, the participants tried to minimize damage by escaping.

#### Susceptibility to critique

“Susceptibility to critique” described being emotionally hurt by the opinions and evaluations of others. Answers that showed participants’ tendency to feel hurt by others’ opinions included “I am directly told bad things about myself” and “my personality/existence/opinion was denied.”

## The main study

### Purpose

We developed an emotional vulnerability scale based on the 42 semantic units obtained in the preliminary survey, determined the reliability and validity of the scale, and examined basic attributes using the scale.

### Method

#### Participants

We collected data from 361 Japanese university students (186 men and 175 women; mean age = 19.6 ± 0.98 years). Survey participants’ grades were as follows: first year (*n* = 40, 11.1%), second year (*n* = 192, 53.2%), third year (*n* = 102, 28.3%), and fourth year (*n* = 27, 7.5%). The participants were from the following university departments: sports and health sciences (*n* = 136), social welfare (*n* = 80), liberal arts (*n* = 51), literature (*n* = 35), economics (*n* = 17), commerce (*n* = 15), medicine (*n* = 11), engineering (*n* = 5), agriculture (*n* = 5), arts (*n* = 3), education (*n* = 1), and sociology (*n* = 1). Regarding membership status in university and off-campus clubs, the distribution was as follows: club activities (*n* = 175), circle activities (*n* = 45), extracurricular club teams (*n* = 13), and no club membership (*n* = 128). As with the preliminary survey, the exclusion criterion was the diagnosis of a mental disorder; no student fulfilled this criterion. All students fulfilled the inclusion criterion of being active university students who had not taken a leave of absence owing to health, academic, or financial reasons in the past year. The recruitment process was identical to that of the preliminary study.

#### Procedure

This study was conducted from June 2021 to July 2021, when classes were held online because of COVID-19-related restrictions. The survey was also conducted online, using Google Forms, and took approximately 10 min to complete. About 3 weeks after the first survey, 64 participants (32 men and 32 women; mean age = 20.0 ± 1.05 years) extracted from the original 361 participants by random sampling completed the emotional vulnerability scale again to assess test–retest reliability.

#### Ethical considerations

Before administering the questionnaires, the participants were fully informed, in writing and verbally, about the purpose of the survey, that participation was voluntary, and that they would not be disadvantaged if they did not participate in the survey. In addition, it was explained that the survey was anonymous, and the survey results would not be used for purposes other than that of this study. Participation in the survey was taken as consent. This study was approved by the institutional review board of the institution to which the first author is affiliated.

#### Measures

The questionnaire survey consisted of the following measures.

##### Sociodemographic questions

We collected data regarding the university students’ sex, age, grade, undergraduate area of study, and club activities.

##### Draft of the emotional vulnerability scale

A draft of the emotional vulnerability scale was prepared using the 42 items obtained from the preliminary survey. The participants responded on a four-point scale, ranging from 1 = “I completely disagree” to 4 = “I completely agree.” The total score was calculated by adding the average of each item. Higher scores indicated higher levels of vulnerability.

##### Scale for measuring depressive symptoms

The self-rating depression scale (SDS) developed by Zung (1965) was used. It consists of 20 items, with four possible responses ranging from 1 = “A little of the time” to 4 = “Most of the time.” Higher scores indicated more severe depressive symptoms. Zung (1965) has reported a split-half reliability of 0.73 for the scale. In the present study, the scale showed good reliability, with a Cronbach’s alpha value of 0.86.

#### Data analysis

In developing the scale, factor analysis was performed in this study based on Wood and Boyce’s (2017) methodology. An exploratory factor analysis (maximum likelihood method/Promax rotation) was performed to determine the factor structure of the emotional vulnerability scale, after which confirmatory factor analysis was performed. Goodness-of-fit index (GFI), adjusted GFI (AGFI), comparative fit index (CFI), and root mean square error of approximation (RMSEA) were used for each GFI. The variance of each latent variable and each path from the error variable to the observed variable was constrained to 1. For reliability, the α coefficient was calculated to confirm the internal consistency, and the intraclass correlation coefficients (ICC) were calculated as a re-examination method at intervals of 3 weeks. Regarding validity, the correlation between vulnerability and depression was determined. Previous studies have reported a link between vulnerability and depressive symptoms (Yamaguchi et al., 2018, 2019). Therefore, this study also treated depressive symptoms as a component of validity. This corresponds to the notion of “Show Criterion Validity” mentioned by Wood and Boyce (2017). After ensuring reliability and validity, demographic data were analyzed using the emotional vulnerability scale. Specifically, we performed a *t*-test to determine sex differences. In the analysis, we decided to make a comprehensive judgment, including the index of effect size (*η*^2^ and partial *η*^2^), instead of examining only the *value of p* indicating the significance level. Regarding partial *η*^2^, there is no clear standard for indicating the magnitude of the effect (Cohen, 1988), but when *η*^2^ is used, it is affected by the number of independent variables and samples. After controlling for the influence of these factors, we also obtained the partial *η*^2^ that calculates the effect size of the influence of one independent variable. IBM SPSS 27.0 and AMOS 27.0 were used for the analyses.

### Results

To confirm the validity of the data for the 42 draft items, the Kaiser–Meyer–Olkin (KMO) and Bartlett Spherical Shape (BS) tests were performed. The KMO measure was.90, and the BS was 2584.050 (*p* < 0.001, *df* = 120).

#### Exploratory factor analysis

For the exploratory factor analysis, the criteria for analysis were eigenvalues ≥1.0 and factor loadings ≤0.40. Consequently, 16 items from four factors were extracted. The four factors were “Vulnerability toward criticism or denial,” “Vulnerability toward worsening relationships,” “Vulnerability toward interpersonal discord,” and “Vulnerability toward procrastination and emotional avoidance” (Table 1).

**Table 1:** Results of the exploratory factor analysis.

Subscale	F1	F2	F3	F4	Communality
F1: Vulnerability toward criticism or denial (*α* = 0.72)					
I get hurt when my opinion is criticized	0.870	0.055	0.010	−0.150	0.696
I get hurt when my thoughts are denied	0.796	0.060	−0.007	−0.054	0.637
I get hurt when someone criticizes me	0.705	−0.098	0.045	0.138	0.575
I get hurt when someone advises me	0.596	−0.043	0.046	0.186	0.511
F2: Vulnerability toward worsening relationships (*α* = 0.79)					
I do not want to be hated, so I feel hurt if I cannot decline an invitation	−0.091	0.849	0.054	−0.026	0.650
I am afraid of being hated by people, and I feel weak and hurt for accepting requests	0.067	0.743	−0.024	0.088	0.691
I feel hurt if I cannot refuse what people have asked me to do	−0.043	0.681	0.020	0.000	0.443
I feel weak and hurt when I cannot oppose people’s ideas	0.211	0.527	−0.058	0.030	0.437
F3: Vulnerability toward interpersonal discord (*α* = 0.80)					
I get hurt when I am directly told bad things about myself	0.001	0.018	0.913	−0.061	0.802
I get hurt when I am indirectly told bad things about myself	0.062	0.057	0.801	−0.126	0.657
I get hurt when my relationship with my friends goes bad	−0.057	−0.057	0.575	0.235	0.420
I get hurt when someone I trust does not talk to me	0.095	−0.020	0.404	0.170	0.313
F4: Vulnerability toward procrastination and emotional avoidance (*α* = 0.79)					
I feel vulnerable when I try to avoid things I do not like	0.076	−0.017	−0.074	0.710	0.504
I feel hurt avoiding things that cause inconvenience to me	−0.081	0.042	0.067	0.688	0.497
I feel regret and hurt when I turn my back toward a problem	−0.020	0.191	−0.026	0.674	0.615
I feel hurt when I put off things I do not like	0.012	−0.063	0.063	0.593	0.350
Cumulative contribution ratio (%)	36.8	45.3	50.6	55.0	

#### Reliability

The α coefficient of each factor was used to determine reliability. The values were as follows: for “Vulnerability toward criticism or denial,” *α* = 0.85; for “Vulnerability toward worsening relationships,” *α* = 0.82; for “Vulnerability toward interpersonal discord,” *α* = 0.81; and for “Vulnerability toward procrastination and emotional avoidance,” *α* = 0.79.

In addition, about 3 weeks after the first survey, a re-examination was performed with 64 participants (32 men and 32 women; mean 20.0 years old, *SD* = 1.05) extracted from the original 361 participants by random sampling. The ICC value for each factor ranged from *ri* = 0.61–0.68 (*p* = 0.001).

#### Confirmatory factor analysis

To investigate the validity of the factors extracted by the exploratory factor analysis, a confirmatory factor analysis was performed. The results showed that the paths from the assumed latent variables to the observed variables, and the path coefficients between the latent variables, were all significant at the 0.1% level, and the model’s GFI was also good (GFI = 0.94, AGFI = 0.91, CFI = 0.96, RMSEA = 0.05).

#### Concurrent validity

Pearson’s product–moment correlation coefficient was calculated for the relationship between depressive symptoms and vulnerability to analyze concurrent validity. A moderate positive correlation was found between depressive symptoms and total vulnerability score (*r* = 0.43, *p* = 0.01). The results showed a significant relationship between the four subscale scores of the emotional vulnerability scale and depressive symptoms (*r*s = 0.25–0.37, *p* = 0.01).

#### Analysis of demographic data

The basic demographic attributes of the participants were examined using the scale created in this study. Women had significantly higher scores than men on both the total emotional vulnerability scale and subscales other than “Vulnerability toward worsening relationships” (Table 2).

**Table 2:** Sex differences in total vulnerability scores and subscale scores (*t*-test).

	Men (*n* = 186)		Women (*n* = 175)		*t*	*p*	Cohen’s *d*
	*M*	*SD*	*M*	*SD*			
Total score of vulnerability	2.6	0.56	2.8	0.53	3.84	0.001	0.40
Vulnerability toward criticism or denial	2.5	0.72	2.8	0.77	4.20	0.001	0.44
Vulnerability toward worsening relationships	2.2	0.71	2.2	0.70	1.10	0.273	0.11
Vulnerability toward interpersonal discord	3.0	0.73	3.3	0.60	4.18	0.001	0.45
Vulnerability toward procrastination and emotional avoidance	2.6	0.69	2.7	0.69	2.48	0.013	0.26

Cohen’s *d*: small = 0.20, medium = 0.50, large = 0.80 (Cohen, 1988).

## Discussion

This study developed an emotional vulnerability scale and examine its basic attributes. The emotional vulnerability scale developed in this study comprises four factors and 16 items and measures the vulnerability of university students in relation to everyday situations and common experiences. In terms of reliability, the obtained coefficients were as follows: “Vulnerability toward criticism or denial,” *α* = 0.85; “Vulnerability toward worsening relationships,” *α* = 0.82; “Vulnerability toward interpersonal discord,” *α* = 0.81; and “Vulnerability toward procrastination and emotional avoidance,” *α* = 0.79. In addition, in the re-examination conducted with 64 participants approximately 3 weeks after the first survey, the ICC values were *ri* = 0.68, 0.61, 0.64, and.66 for the first, second, third, and fourth factors, respectively, all of which were significant at the 0.1% level. The ICC values of 0.61–0.80 are considered constant and 0.81–1.00 are considered almost perfect (Landis and Koch, 1977). In this study, *ri* values were.61–0.68; thus, they were substantial. The GFI of the model assessed using the confirmatory factor analysis was GFI = 0.94, AGFI = 0.91, CFI = 0.96, and RMSEA = 0.05. When the obtained values are applied to the criteria, a GFI of 0.90 or higher, a CFI of 0.95 or higher, an AGFI of 0.90 or higher, and an RMSEA of.05 or lower are regarded as an acceptable fit (Schermelleh-Engel et al., 2003). Therefore, all ICC and numerical values of the GFI of each model obtained in this study met the criteria. According to Wood and Boyce (2017), ideally, 450 participants are required for factor analysis (and at least 150 or more); this study involved 360 participants. The sample size is a little less than 100 short of the standard 450 people; however, the analysis shows that the reliability and validity are high. Thus, we think that this scale is reliable and effective.

The concept of vulnerability investigated in this study was proposed by Sinclair and Wallston (1999), and the measure of vulnerability developed by them, the PVS, has been often used in existing studies. Schaufeli et al. (2002) indicate the importance of creating a measure tailored to the characteristics (occupation, sex, etc.) of the participants and based on an accurate psychological assessment when preparing the questionnaire. Although the scale developed by Sinclair and Wallston (1999) has been used widely, this scale was created for patients with rheumatoid arthritis. Therefore, it is likely that the scale focuses on the difficulties in life experienced by patients with rheumatism, and it may not be possible to rule out the factors of vulnerability specific to this health condition.

We also used the developed scale to examine its association with depressive symptoms. A positive correlation was found between vulnerability and depressive symptoms. Since this result is consistent with previous research (Hayashi, 2002; Yamaguchi et al., 2018), we believe that the measure developed in this research reflects the construct of vulnerability. Therefore, if one is vulnerable to something happening in one’s daily life, one may experience more depressive symptoms. Prior studies have shown the association of vulnerability with not only mental health (Hayashi, 2002; Yamaguchi et al., 2019) but also social connections (Dang, 2014). Specifically, a lack of social connections may lead to psychological vulnerability, which could contribute to poorer mental health outcomes (Dang, 2014). Moreover, psychological vulnerability is negatively correlated with resilience factors such as social support and self-efficacy (Kiamarsi and Abolghasemi, 2014; Satici et al., 2014; Gruebner et al., 2015). From these trends, we believe that the same results as those of the abovementioned studies can be obtained even with a scale that explores vulnerabilities among university students, and not just the PVS, which was developed for rheumatism patients. Additionally, Satici et al. (2016) revealed a negative relationship between psychological vulnerability and social safety and found social safety to be a significant negative predictor of psychological vulnerability. Additionally, similar to the findings of Yamaguchi et al. (2018, 2019), who studied the relationship between vulnerability and mental health, Demirci et al. (2019) reported that psychological vulnerability is an important factor for mental health and well-being. According to Yelpaze et al. (2021), empirical support for potential factors in the relationship between psychological vulnerability, social connectedness, and well-being is still lacking. This study does not explore any association with social ties or safety. However, vulnerable people may be unable to block relationships or use social support well to cope with their trauma. Hence, research on vulnerability requires further development. The subfactors of the scale developed in the current study are described below.

The first factor, “Vulnerability to criticism or denial,” included items such as “I get hurt when my opinion is criticized” and “I get hurt when someone criticizes me.” It comprises content that expresses excessive hurt as a result of reactions such as being criticized by others. Adolescents are more anxious about negative evaluations from others (Westenberg et al., 2004). According to Leary et al. (1998), “the perception that one is underestimated by others” is highlighted as a hurtful feeling in interpersonal relationships. Therefore, inattention, denial, and criticism by others are considered typical causes of emotional hurt. Such as, hurtful verbal communications are also cited as a factor that causes feelings of psychological hurt (Vangelisti, 1994). For example, verbal expressions such as “Going out with you was the biggest mistake of my life” and “You’re such a hypocrite.” Therefore, some people are expected to be overly pained by the remarks of others. Thus, a high score on “Vulnerability to criticism or denial” means that the person experiences excessive psychological hurt by the words and actions of other people.

The second factor, “Vulnerability toward worsening relationships,” is related to interpersonal relationships. It includes items such as “I do not want to be hated, so I feel hurt if I cannot decline an invitation” and “I feel weak and hurt when I cannot oppose people’s ideas.” It involves attempts to delicately repair damage so that the relationship does not deteriorate and thus avoid the resultant pain. Rejection as a member of a group by others may reduce self-esteem (Leary et al., 1995) and increase self-damaging behavior (Williams et al., 2000). Therefore, it is conceivable that vulnerable individuals may experience increased hesitation to nurture relationships or increase their self-defeating behaviors and beliefs when rejection occurs. Additionally, unavoidable reactions to avoid rejections may exacerbate the issue. Thus, a high score on “Vulnerability toward worsening relationships” indicates being hurt by trying to build a relationship at the expense of one’s own time and convenience.

The third factor, “Vulnerability toward interpersonal discord,” refers to the worsening of interpersonal relationships as expressed by items such as “I get hurt when I am directly told bad things about myself” and “I get hurt when someone I trust does not talk to me.” Slander from a person is an example of this factor. Backbiting can be regarded as bullying, although the person who engages in it may think that it is a “joke.” Insults, name-calling, derogatory or humiliating comments, embarrassment, exclusion from the group, backbiting, and events that disrupt friendship are considered “emotional bullying” (Arslan Özdinçer and Savaşer, 2008). Therefore, those who are vulnerable will take such jokes at face value, and it is expected that they will be further hurt, similar to the feeling of being bullied. As explained in the discussion of the second factor, vulnerable people tend to care about the views of others. Exclusion from the group in interpersonal relationships is an attack on the relationship, which reportedly increases anxiety, loneliness, and depression and seriously damages the relationship (Gazelle and Ladd, 2003). Thus, “Vulnerability toward interpersonal discord” refers to a state of being hurt when someone speaks badly of oneself, when the relationship with the other becomes unsatisfactory.

The fourth factor, “Vulnerability toward procrastination and emotional avoidance,” includes items such as “I feel vulnerable when I try to avoid things I do not like” and “I feel hurt when I put off things I do not like.” It comprises content that indicates someone is hurt excessively when there is a problem but suppresses the degree of hurt by escaping the event or problem and running away, which results in them being hurt. Escapist coping strategies have been shown to increase burnout when something goes wrong (Leiter, 1991). Therefore, if one faces a problem and tries to deal with it, one will be hurt, however, avoidance could carry the same risk. In addition, those who are vulnerable will be hurt by both actions, by either dealing with or avoiding the problem; moreover, if they avoid or escape the situation, they will blame themselves for being in the situation in the first place.

Leary et al. (1998) asked college students to freely describe vulnerable events. “Active separation,” “blame,” “betrayal,” “teasing,” “not cherished,” and “disregarded” were expressed as hurtful events. According to Vangelisti (1994), “evaluation,” “blame,” “instruction,” “joke,” “threat,” and “doubt” are listed as factors and situations that caused pain. Based on these facts, the contents of each factor extracted in this study generally support the vulnerable events extracted in past studies, and the scale developed in this study covers vulnerable events in daily life.

Next, sex differences were investigated. We found that women scored significantly higher in terms of vulnerability than men. Women were more vulnerable than men. This result is similar to that of previous studies (Hayashi, 2002; Yamaguchi et al., 2019), suggesting that the scale developed in the present study yields an accurate assessment of vulnerability. However, when examining each factor, the only factor in which sex differences were not confirmed was “Vulnerability toward worsening relationships.” The university students surveyed were in the same peer group and therefore in the same stage of psychological development. Peer groups are considered to be a group in which members share each other’s values and ideals, recognize each other’s differences, and respect each other as independent individuals. Among these groups, same-sex friendships significantly support adolescents (Bagwell et al., 2005); in adolescence, these relationships are extremely intimate and may involve friends of the same age (Sullivan, 2013). Not comparing themselves to others and believing that each person has their own place in the world may help college students refuse unwelcome invitations and requests. However, this was not observed in the present study; moreover, it is conceivable that vulnerable individuals will accept any invitation or request out of fear of damaging interpersonal relationships, or they may experience a conflict between not wanting to get hurt and not wanting to hurt others. No sex differences were observed regarding this factor. Furthermore, when peer groups discuss various life situations, sharing positive experiences with each other improves psychological well-being (Demir et al., 2013). However, interpersonal conflicts and others’ negative behavior toward an individual can lead to poor mental health. For example, a study of college athletes (Yamaguchi et al., 2019) found that people with higher vulnerability were more likely to develop depressive symptoms. Although the effect of vulnerability on depressive symptoms has not been investigated in this study, it could be an important research topic to be considered in future studies.

From the above discussion, using the emotional vulnerability scale developed in this study, it is possible to understand participants’ vulnerability in interpersonal relationships and events in daily life. In addition, using this scale can help determine a person’s level of vulnerability and predict possible mental health disorders, as vulnerability is expected to be a precursor to depressive symptoms (Yamaguchi et al., 2018, 2019). In fact, if adolescents experience hurt as threatening, they recall it repeatedly, which leads to increased stress (Joseph and Williams, 2005). Therefore, it can also serve as an important assessment tool in clinical situations. Therefore, it is suggested that understanding individuals’ vulnerabilities will help prevent mental health issues, which is a significant contribution to related efforts in the health psychology field.

### Limitations and further developments

This study has some limitations. A questionnaire survey was used to measure participants’ “emotional hurt.” Participants may have provided false responses to conform to socially acceptable values, avoid criticism, or gain social approval (Huang et al., 1998; King and Brunner 2000). It is conceivable that the evaluation of concepts that can be measured by questionnaire surveys, including one’s “vulnerable state” that can be measured in this research, may change depending on participants’ subjective responses. For example, when measuring “vulnerability,” we believe that measuring social desirability (Van de Mortel, 2008) concurrently can reduce the distortion of the measurement. In fact, according to Van de Mortel (2008), the tendency for people to present a favorable image of themselves on questionnaires is called “socially desirable responding.” Consequently, some people may underestimate the “emotional hurt” and try not to show weakness. Therefore, future researchers should include measures of social desirability.

Second, the SDS scale, used for concurrent validity, assessed only depressive symptoms. While vulnerability is associated with mental health and other stress responses (Yamaguchi et al., 2022), it is also associated with negative personality traits and social desirability. Future research should examine the associations with these concepts, as well.

Third, as this study only involved university students, it is possible that the obtained verbal data and questionnaire items reflect the vulnerability factors experienced only during university. There may be factors unique to each generation in the vulnerabilities of everyday situations that different populations can experience. Presently, the scale created in this study is intended for university students, based on our study’s target group; its applicability, however, is not limited to athletes and rheumatism patients, as is the case with other existing standardized scales, and can be used among the general public. When using this scale with other age groups, it will be necessary to evaluate its reliability and validity. This time, we focused on university students, furthermore, it will be necessary to assess different populations, such as elementary, junior high, and high school students, as well as adults with different attributes, to generalize the results.

Lastly, as this study was a one-point cross-sectional survey, a causal relationship between vulnerability and depression could not be determined. As the impact of COVID-19 has been of great concern and a source of stress for the targeted university students, it will be necessary to conduct longitudinal surveys to determine a causal relationship between vulnerability and stress responses.

## Conclusion

We developed an emotional vulnerability scale, confirmed its reliability and validity, and examined differences among participating university students based on demographic data. A four-factor structure scale was developed, and reliability and validity were assessed. This scale can be used to evaluate the vulnerable emotions and conditions experienced by individuals that cause them pain. Women were more vulnerable than men. The results suggest that the scale can be used to determine the vulnerability level of an individual and that it is effective as an assessment tool for mental health issues. From the viewpoint of health psychology, we believe that the scale could inform efforts toward regulating stress responses and reducing depressive symptoms and could provide opportunities to minimize painful feelings experienced as part of daily life.

## Data availability statement

The original contributions presented in the study are included in the article/Supplementary material, further inquiries can be directed to the corresponding author.

## Ethics statement

The studies involving human participants were reviewed and approved by Juntendo University. Written informed consent for participation was not required for this study in accordance with the national legislation and institutional requirements.

## Author contributions

SY designed the study, collected all the data, performed the statistical analysis, and prepared the manuscript. YK supported the study design and data collection processes. YM and TO provided expert comments for the scale development process according to their specialties. YM contributed to sports medicine. TO contributed to psychiatry. All authors read and approved the final manuscript.

## Funding

This work was supported by the Joint Research Program of Juntendo University, Faculty of Health and Sports Science. Additionally, this work was also supported by JSPS KAKENHI Grant Numbers 19K24291, JP20K19517.

## Conflict of interest

The authors declare that the research was conducted in the absence of any commercial or financial relationships that could be construed as a potential conflict of interest.

## Publisher’s note

All claims expressed in this article are solely those of the authors and do not necessarily represent those of their affiliated organizations, or those of the publisher, the editors and the reviewers. Any product that may be evaluated in this article, or claim that may be made by its manufacturer, is not guaranteed or endorsed by the publisher.
