# Supplementary material for: The development and validation of an emotional vulnerability scale for university students
Source: Front Psychol. 2022 Sep 15;13:941250. doi: 10.3389/fpsyg.2022.941250 (PMC9521647; doi:10.3389/fpsyg.2022.941250)
Supplement: Supplementary file 1 [file Table_1.docx]

Supplementary Material

# Supplementary Tables

Supplementary Table 1. Statements by category

| Main category | Subcategory | Actual answers |
| --- | --- | --- |
| Remorse | I feel hurt when I feel inferior to others. | I feel inferior compared to others |
|  | I feel hurt that I am pessimistic about interpersonal relationships. | I think negatively about human relationships. |
|  | I am depressed if I am not helpful towards others. | I feel powerless when I cannot help a friend who is in trouble. |
|  | I feel sorry for myself when I see a person’s complexion. | It will hurt if you show your feelings on your face |
|  | I am depressed as I carry my mistakes around forever. | I will carry my mistakes around forever. |
|  | I am depressed because I cannot do things well. | I am disappointed in myself when I am not able to solve problems. |
| Diluted relationship | I get hurt when someone I trust does not talk to me. | People I trust do not talk much with me. |
|  | I get hurt when my relationship with my friends goes bad. | I do not get on well with friends. |
|  | I am depressed when I feel misunderstood. | I misunderstand myself. |
|  | I am emotionally hurt when I feel lonely. | I feel alone. |
|  | I am hurt when I feel people do not need me. | I feel like I am out of place. |
|  | I feel sad when my friends respond late. | The other party’s reply is slow. |
| Pressure | I am depressed when I feel responsible for a problem. | I feel sick when I am responsible for something. |
|  | I am depressed when I think about my future. | I feel depressed when thinking about having to do something I do not like. |
|  | I am not confident speaking in public. | Speaking in front of many people makes me nervous. |
|  | I lose confidence when I am in a competition. | I feel powerless after a loss in a game. |
|  | I feel hurt by my mistakes. | I am scared to fail. |
|  | I feel hurt when something unreasonable is said to me. | Unreasonable things are said. |
| Difficulty of refusal | I feel hurt if I cannot refuse what people have asked me to do. | I cannot argue. /I cannot oppose. |
|  | I do not want to be hated, so I feel hurt if I cannot decline an invitation. | I cannot refuse. /I am pitiful if I cannot refuse. |
|  | I am hurt if I cannot convey my thoughts clearly. | I cannot convey my thoughts. |
|  | I am afraid of being hated by people, and I feel weak and hurt for accepting requests. | I do not want to be disliked. /I cannot decline an invitation. |
|  | I do not want to be hated, so I am depressed if I cannot decline an invitation. | I do not want to be disliked. /I cannot decline an invitation. |
|  | I feel weak and hurt when I cannot oppose people’s ideas. | I cannot argue. /I cannot oppose. |
| Procrastination | I am depressed and I do not want to do anything. | I do not want to do anything. |
|  | I have things to do but I feel hurt when I cannot get anything done. | I am tired and cannot do that. /I do not want to do it. /I have to do it, but I do not. |
|  | I am depressed when I am alone. | I do nothing when I am alone. |
|  | I am depressed if I cannot respond to people’s requests. | I cannot help. /I cannot live up to my expectations. |
|  | I am depressed when everything I do feels like a bother. | What I do feels bothersome. |
|  | I am depressed when I cannot do anything because I lack skills. | I have no skills. /I cannot help. |
| Avoidance/escape | I feel vulnerable when I try to avoid things I do not like. | I want to run away. /It is not convenient for me. |
|  | I feel hurt when I put off things I do not like. | I will procrastinate. /I give up. |
|  | I feel hurt avoiding things that cause inconvenience to me. | I cannot keep it going. |
|  | I feel depressed because I feel my intention is weak. | I have a weak will. |
|  | I feel regret and hurt when I turn my back towards a problem. | I disregard the problem. I run away from the problem. |
|  | I get hurt when someone advises me. | I was criticized. |
| Critical evaluation | I get hurt when I am directly told bad things about myself. | I am directly told bad things about myself. |
|  | I get hurt when I am indirectly told bad things about myself. | I was told things indirectly. |
|  | I lose confidence when my humanity is denied. | My personality was denied. / Existence was denied. / Opinion was denied. |
|  | I get hurt when my thoughts are denied. | My personality was denied. / Existence was denied. / Opinion was denied. |
|  | I get hurt when my opinion is criticized. | My personality was denied. / Existence was denied. / Opinion was denied. |
|  | I get hurt when someone criticizes me. | I was criticized. |
